# Joint Action Enhances Cohesion and Positive Affect, but Suppresses Aspects of Creativity When Combined With Shared Goals

**DOI:** 10.3389/fpsyg.2018.02790

**Published:** 2019-01-15

**Authors:** Reneeta Mogan, Joseph Bulbulia, Ronald Fischer

**Affiliations:** ^1^Mind, Body, Cultural Evolution Laboratory, School of Psychology, Victoria University of Wellington, Wellington, New Zealand; ^2^School of Humanities, Faculty of Arts, The University of Auckland, Auckland, New Zealand

**Keywords:** synchrony, creativity, convergent thinking, divergent thinking, shared intentionality, cohesion, positive affect, cultural evolution

## Abstract

We aimed to examine the link between two types of joint action (synchrony and asynchrony) and creativity (both divergent thinking and convergent thinking) using an established experimental paradigm. A secondary aim was to replicate and extend the amplified positive effects of shared intentionality (i.e., having a shared common goal) on social and affective responses. Participants (*N* = 138) were randomly assigned to move in synchrony, move in asynchrony, or passively observe others moving. To induce shared goals, participants were provided with either a shared group goal of working together or an individual goal of focusing on the individual participant’s own movements. First, our results revealed that joint action in combination with group goal conditions decreases convergent thinking, but we found no support for differences in divergent thinking. This indicates that it may be the underlying shared goals combined with joint action that influences convergent thinking, and not synchronized movements. Second, we replicated synchrony’s positive effect on cohesion and positive affect. These findings are consistent with evolutionary theories of group rituals as a means for inducing solidarity, and extend previous findings by showing that joint action with shared goals may potentially induce shared patterns of thought.

## Introduction

Rituals have been a fundamental part of human life for millennia possibly due to tacit evolutionary functions of increasing solidarity and cooperation (Hagen and Bryant, 2003; Haidt et al., 2008; Henrich, 2015). A key element of ritualistic behavior is synchrony – the matching of actions in time with others (Hove and Risen, 2009). Widespread synchronized actions in human culture have been associated with numerous benefits, including increased cooperation, trust, cohesion, and positive affect among group members (Wiltermuth and Heath, 2009; Reddish et al., 2013; Tschacher et al., 2014; Mogan et al., 2017). These effects appear to be amplified when people share a common goal (Reddish et al., 2013), which supports Tomasello et al.’s (2005) suggestion that shared intentionality (having a shared goal) is a crucial component of human evolution and the emergence of complex cultural traditions.

In addition to rituals increasing solidarity and cooperation, another function of rituals is to regulate social connections through the transmission and maintenance of social norms to manage conflicts and preserve cohesion by decreasing individual differences (Lienard and Boyer, 2006; Rossano, 2012; Hobson et al., 2017). If synchrony were a key element of collective rituals, then synchronous actions would act to dampen individuality as well. Hence, while synchrony may be beneficial in some circumstances (i.e., to induce positive social and affective effects), it may be potentially detrimental in circumstances requiring more individuality and innovation rather than convergence to cultural group norms.

Creativity – the ability to produce ideas and responses which are new, appropriate, and useful to problems (Amabile, 1983) – is one of the most common ways of expressing individuality (Dollinger et al., 2007; Bechtoldt et al., 2012). Guilford (1967) categorized creativity into divergent thinking (generating multiple alternative and typically original solutions to a problem) and convergent thinking (forming logical associations to synthesize information to generate one best and accurate solution to a problem, see Cropley, 2006). Synchrony may affect these two creativity components differently.

We speculate that synchrony reduces individuality and independent thought, which should lead to reduced divergent thinking, but may increase convergent thinking. Durkheim (1912/1995) conjectured that matching movements during rituals leads to conformity of thought. Evidence suggests that matching someone’s actions and observing those matching actions blurs self and other perceptions in the mind of the participating members (Hove, 2008; Hurley, 2008). This increases perceptions of similarity (Reddish et al., 2013; Dong et al., 2015; Liebenberg, 2017), which potentially results in more convergence and conformity in thought processes and shared mental states (Baimel et al., 2015), thus facilitating convergent thinking. This mental state could also stifle uniqueness and individuality, thereby inhibiting divergent thinking. This reasoning is in line with real-world observations on highly cohesive and similar groups having poorer decision-making quality (i.e., the groupthink phenomenon; Janis, 1982). We are the first to experimentally test these predictions.

A secondary aim of this study was to replicate and extend previous work on shared intentionality (i.e., shared goals) as a possible underlying mechanism explaining synchrony’s positive effect on social and affective responses. Examining the relationship between shared goals on creativity is important because shared goals appear to be a crucial factor leading to more conformity of thought (Hu et al., 2017). Therefore, extending Reddish et al.’s (2013) experimental paradigm, we predicted greater convergent thinking and less divergent thinking when shared goals are manipulated. We also report cohesion and affective outcomes, to examine the replicability of synchrony effects on social and affective outcomes (see Mogan et al., 2017).

## Methods

### Participants

One hundred and thirty-eight students (*M* = 19 years, *SD* = 3.33 years; 88 females) were randomly assigned to groups of three at a time, in exchange for course credits. The number of participants in each cell ranged from 21 to 30 participants. The study was approved by the university’s Human Ethics Committee (Ref: RM019282), and all participants provided written consent.

### Design and Procedure

We used a 3 (Movement: synchrony, asynchrony, passive observation) × 2 (Goal: individual, group) between-subjects factorial design. We used the stepping paradigm by Reddish et al. (2013, experiment 3). Participants in the synchrony and asynchrony (joint action) conditions were each given a pair of headphones, through which metronome beats were played. They were instructed to march on the spot for 6 min by stepping their foot down on each beat. They were also instructed to move the same side arm (i.e., left arm with left leg) up and down while stepping.

Participants in the synchrony condition performed the movements in time with each other. In the asynchrony condition, they each performed the movements at different speeds. In the passive condition, participants silently watched a video of three confederates performing the movements in synchrony for 6 min.

In the individual goal conditions, participants were told to only pay attention and move according to the metronome beats heard through their headphones, which were played for the full 6 min. In the group goal conditions, the metronome beats were played for the first 30 s. After the sound stopped, participants were instructed to work together to perform the movements according to their assigned movement condition. Participants in the synchrony condition were instructed to work together to move in time with each other. Participants in the asynchrony condition were instructed to work together to move out of time with each other.

Participants were not informed of the synchrony hypothesis prior to the study. Upon completion of the study, participants were funnel debriefed. No participant accurately identified the purpose of the study. They were then presented a debriefing sheet explaining the synchrony hypothesis. Full instructions and power analyses are available on Fischer and Mogan’s (2018) Open Science Framework [osf.io/9rcze].

### Dependent Variables

We measured creative thinking performance immediately after the movement manipulation. The two tasks were counterbalanced across individuals. We used the Alternate Uses Task (AUT; Guilford, 1967) to assess divergent thinking. Participants had to list as many uses as they could for two items – newspaper and paperclip, and were given 2 min for each item. Two independent coders rated each response on creativity (intra-class correlation [ICC] = 0.78, 95%CI [0.76, 0.80]), and novelty (ICC = 0.71, 95%CI [0.69, 0.74]) on a 7-point Likert scale (Consensual Assessment Technique; Amabile, 1982). We included fluency (each participant’s total number of responses) as a third index. For convergent thinking, participants had 2 min to solve 10 items of the Remote Associates Test (RAT; Mednick, 1962). Each item contained a set of three unrelated clue words (e.g., “light,” “birthday,” “stick”), and the task was to identify a fourth conceptually related word (e.g., “candle”).

Cohesion was measured as a composite of four constructs adapted from Reddish (2012; interconnectedness, entitativity, trust, and perceived similarity, Cronbach’s α = 0.91). We used the Positive and Negative Affect Schedule (PANAS; Watson et al., 1988) to assess participants’ positive and negative affect. Six positive affect items (α = 0.79), and five negative affect items (α = 0.73) were rated on a 7-point Likert scale.

Finally, to control for familiarity, we asked participants to rate how well they knew each participant before they met for the study. We found a significant effect, *F*(2, 132) = 6.43, *p* = 0.002, η_p_^2^ = 0.09, therefore all analyses are reported controlling for familiarity by including familiarity as a covariate in the Analyses of Covariance (ANCOVA).

## Results

### Creative Thinking

#### Divergent Thinking

The results showed no statistically significant mainor interaction effects of movement and goal, *F*_max_ = 1.37, *p* = 0.257, on fluency, creativity, and novelty.

#### Convergent Thinking

**FIGURE 1:**
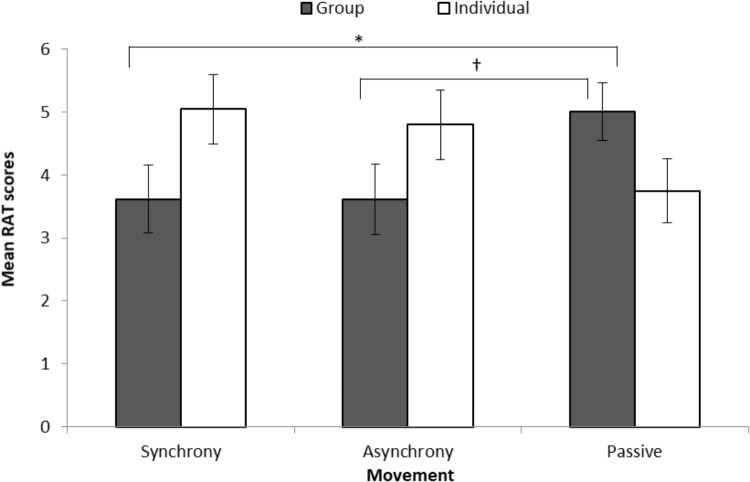
Remote associates test adjusted mean scores based on movement and goal with standard errors. ^∗^*p* = 0.054; ^†^*p* = 0.061.

We did not find a statistically significant main effect of movement, *F*(2, 131) = 0.05, *p* = 0.947, η_p_^2^ < 0.01, or goal, *F*(1, 131) = 1.14, *p* = 0.287, η_p_^2^ = 0.01, on the RAT. However, we found a statistically significant Movement x Goal interaction, *F*(2, 131) = 4.31, *p* = 0.015, η_p_^2^ = 0.06. LSD-corrected pairwise comparisons revealed a marginally significant difference between movement conditions in the group goal condition, *F*(2, 131) = 2.58, *p* = 0.079, η_p_^2^ = 0.04. Figure 1 shows that participants in the synchrony condition (*M* = 3.62, *se* = 0.54) had significantly lower convergent thinking scores than the passive condition (*M* = 5.01, *se* = 0.46, *p* = 0.054). Participants in the asynchrony condition (*M* = 3.61, *se* = 0.56, *p* = 0.061) also had marginally lower scores than the passive condition. We did not observe a statistically significant difference between movement conditions in the individual goal condition, *F*(2, 131) = 1.74, *p* = 0.179, η_p_^2^ = 0.03, but Figure 1 shows a trend that performing synchronous and asynchronous actions with an individual goal may facilitate convergent thinking compared to passive observation.

### Cohesion and Affect

We found a statistically significant main effect of movement on cohesion, *F*(2, 131) = 3.84, *p* = 0.024, η_p_^2^ = 0.06, and positive affect, *F*(2, 131) = 3.88, *p* = 0.023, η_p_^2^ = 0.06. No other statistically significant differences were found, *F*_max_ = 2.11, *p* = 0.125.

Table 1 shows that for cohesion, LSD-corrected pairwise comparison revealed cohesion ratings were statistically significantly higher in the synchrony condition (*M* = 3.72, *se* = 0.11) compared to the passive condition (*M* = 3.31, *se* = 0.10, *p* = 0.006). The synchrony and asynchrony conditions (*M* = 3.52, *se* = 0.11, *p* = 0.194) did not significantly differ.

**Table 1:** Adjusted means and standard errors (in parentheses) of each condition.

Dependent variables	Synchrony		Asynchrony		Passive	
	Group	Individual	Group	Individual	Group	Individual
Fluency (DT)	14.09 (1.09)	12.41 (1.09)	14.19 (1.12)	12.81 (1.10)	13.76 (0.92)	14.74 (1.02)
Creativity (DT)	3.47 (0.14)	3.53 (0.14)	3.20 (0.14)	3.35 (0.14)	3.31 (0.12)	3.39 (0.13)
Novelty (DT)	3.77 (0.13)	3.91 (0.13)	3.54 (0.13)	3.72 (0.13)	3.73 (0.11)	3.72 (0.12)
Convergent thinking	3.62 (0.54)	5.05 (0.55)	3.61 (0.56)	4.80 (0.55)	5.01 (0.46)	3.75 (0.51)
Cohesion	3.63 (0.16)	3.82 (0.16)	3.56 (0.16)	3.47 (0.16)	3.35 (0.13)	3.28 (0.15)
Positive affect	3.56 (0.23)	3.58 (0.23)	3.29 (0.24)	3.65 (0.23)	3.04 (0.20)	2.96 (0.22)
Negative affect	1.50 (0.16)	1.52 (0.16)	1.62 (0.17)	1.58 (0.16)	1.82 (0.14)	1.80 (0.15)


*The three indices of divergent thinking are denoted with a “DT” in parantheses.*

Supporting Mogan et al.’s (2017) meta-analysis that coordinated action increases positive affect, the synchrony (*M* = 3.57, *se* = 0.16) and asynchrony (*M* = 3.47, *se* = 0.17) conditions did not statistically significantly differ from each other (*p* = 0.674), but were significantly (synchrony: *p* = 0.010; asynchrony: *p* = 0.042) higher than the passive condition (*M* = 3.00, *se* = 0.15). Together, our results indicated that performing actions together with others is sufficient to increase cohesion and positive affect among members, regardless of shared intentions.

## Discussion

### Joint Action’s Effect on Creativity

Joint action affects convergent thinking, but it depends on participants holding shared goals. When provided with a shared group goal, individuals performing synchronous and asynchronous actions showed impaired convergent thinking processes, whereas individuals passively watching performed significantly better. Overall, these findings were not in the direction that we had expected.

A possible explanation is reduced cognitive capacity in a state of shared goals due to the increased attention required to successfully move in time (synchrony) or out of time (asynchrony) with each other without the help of a metronome beat (Reddish et al., 2013). This may have led to lower convergent thinking scores for participants in the active movement conditions compared to those who passively observed movements. In other words, paying attention to others may be cognitively demanding and therefore reduces cognitive capacities available for subsequent cognitive tasks.

Using this line of reasoning, we should also find significant effects for divergent thinking scores. Yet we did not find the same pattern, indicating that specific movements or goals do not affect one’s ability to generate multiple ideas. It may be that the duration of the movement activity was too short to elicit effects on divergent thinking. In their meta-analysis, Chang et al. (2012) found that physical activity of at least 20 min was necessary to observe effects on cognitive processes. Divergent thinking may be one of the cognitive functions that requires a longer duration of movement activity. Cognitive effects might be weaker and may require more long lasting interventions. Yet, overall our manipulation was successful as previously reported effects on cohesion and affect were replicated (see below).

As a reviewer noted, many previous studies on synchrony (e.g., as in Koole and Tschacher’s 2016 review in Frontiers) differed in one decisive point from this study: they measured synchrony as a spontaneous phenomenon in naturalistic settings, not as a prescribed laboratory task. It is possible that synchrony’s effects depend crucially on whether it emerges spontaneously outside participants’ awareness or is instructed. Asynchrony as demanded by a laboratory task is qualitatively different from being out of synchrony in a naturalistic interaction.

### Joint Action’s Effects on Cohesion and Positive Affect

We replicated established findings from recent studies (Mogan et al., 2017; Baimel et al., 2018). Both synchronous and asynchronous actions increased cohesion and positive affect among participants compared to participants who passively observed synchronous actions. Joint action as a whole, regardless of exact synchronicity, appears to be sufficient to elicit positive social and affective effects. In support of Durkheim (1912/1995) concept of *collective effervescence* and Turner’s (1969) *communitas*, when people come together to move together, they form a bond which translates into positive social and affective outcomes for participating members involved. We added a passive control condition. Our results suggest that these positive effects do not translate to passive observers. This is an important observation, particularly given reports of observer effects in previous field studies (Konvalinka et al., 2011; Xygalatas et al., 2013).

## Conclusion

Our study is the first to examine the direct association between joint action and creativity. We drew upon sociological analyses which suggest that synchrony’s positive effect on social outcomes may affect creativity by facilitating convergent thinking and impairing divergent thinking. We did not find support for this hypothesis. Nevertheless, our study offers a preliminary notion that it may not be synchronized movement as such, but rather the underlying shared goals that may influence convergent thinking. Synchrony effects on cohesion and positive affect were replicated. We believe our finding that joint action in the context of shared goals suppresses aspects of creativity, offers support for Durkheim’s conjecture about the effects of ritualized behaviors. We note that many naturally occurring rituals are choreographed rather than a strictly enforced matching of behaviors in time. This result may have theoretical implications for the evolution of rituals by revealing a place for choreography as well as synchrony.

## Ethics Statement

This study was carried out in accordance with the recommendations of the Victoria University of Wellington’s Human Ethics Committee with written informed consent from all subjects. All subjects gave written informed consent in accordance with the Declaration of Helsinki. The protocol was approved by the Victoria University of Wellington’s Human Ethics Committee.

## Author Contributions

RM, JB, and RF developed the study concept and design. RM conducted the data collection and data analysis, and drafted the manuscript. JB and RF provided critical revisions. All authors approved the final version of the manuscript for submission.

## Conflict of Interest Statement

The authors declare that the research was conducted in the absence of any commercial or financial relationships that could be construed as a potential conflict of interest.
